# Supplementary material for: Supervision, Moral Distress and Moral Injury Within Palliative Care—A Qualitative Study
Source: Int J Environ Res Public Health. 2025 Jul 21;22(7):1156. doi: 10.3390/ijerph22071156 (PMC12295009; doi:10.3390/ijerph22071156)
Supplement: Supplementary file 1 [file ijerph-22-01156-s001.zip › ijerph-3707449-supplementary.pdf]

Guide to the interviews

The interview guide will be applied flexibly, depending on the individual focus of each interviewee.

Data Protection

If you agree, I will now begin the interview and start the audio recording.

Before we begin, I would like to briefly inform you about data protection. All data from the interview that could be linked to your identity will be pseudonymized, meaning it will not be possible to trace it back to you personally. Your information will be treated with strict confidentiality. All members of the research team are bound by confidentiality obligations. Data will not be passed on to third parties.

Participation in this study is voluntary. You may end your participation at any time and without giving reasons, or withdraw your consent. If requested, your interview recording will be permanently deleted and will not be used.

If you have any questions about data protection, please feel free to ask.

Introduction to the interview and opening question (main narrative)

How are you feeling today? Is there anything you would like to share before we begin the interview?

Please briefly tell me again what professional qualification you have and how long you have been working in this field.
Before we begin discussing the topic of "Moral Injury" in more detail, I would like to ask you first to talk about your current work-related stress. We will later refer back to this as we proceed.

Instructions: While you are speaking, I will refrain from making comments or follow-up questions and give you plenty of time to talk. I will only take a few notes and come back to them later. We have enough time. If you need a break, please let me know.

Prompt to tell a story: I'm interested in hearing your first thoughts that come to mind regarding your current work-related stress.

Possible follow-up questions:

- Which stress factors would you consider the most serious?

- What has been your experience with working overtime?

Please think back to situations when your workload was very high. Tell us about your experiences in this context. Take as much time as you like and go into any details that you feel are important.

Depending on the situation described, use the following guiding questions: How did you perceive this situation, and what would you have wished for? Did you express your concerns to colleagues or supervisors? What were their responses?

Narrative prompt:
In the information flyer, the term "Moral Injury" was already mentioned briefly—meaning a form of "moral violation" or "moral harm." Could you describe what this term means to you from your perspective?

Now I would like to briefly explain the phenomenon of “Moral Injury” and then hear your thoughts. Please take your time to reflect as needed.

For many years, the number of health care workers diagnosed with burnout has been rapidly increasing, and treatments or preventative measures have not led to the hoped-for success. Strategies like resilience can be helpful in the case of burnout, as they strengthen individual perspectives and promote a balanced approach to work and personal life. In this way, one’s own robustness and resistance to stressful circumstances can be developed and solidified.
The opposite of resilience, then, is a state of vulnerability or being hurt. When it became clear that this invulnerability and human resilience could not be fully achieved through the usual protective or resilience-enhancing factors—as is often the case with burnout—scientists began to suspect a link to the phenomenon of “Moral Injury.” This term originally emerged from the experiences of U.S. soldiers returning from war zones.
Due to the COVID-19 pandemic, “Moral Injury” gained more attention in the healthcare sector, as medical professionals—especially nurses—had to work at the limits of their personal capacities and were increasingly confronted with morally and ethically questionable decisions or observations that conflicted with their own moral and ethical

beliefs. These stressors often originate from systemic issues, such as legal regulations or structural conditions, which individuals can only influence to a limited extent.

Narrative prompt:
Can you generally imagine situations in which Moral Injury may occur? These don't have to be your own experiences; they could also be things colleagues have experienced.

I would now like to give you a more in-depth example:
A powerful example of "Moral Injury" is the account of a nurse who worked in an intensive care unit during the peak of the pandemic. She reported having to urgently treat a ventilated patient in one room, while in the next room, a patient was dying without being allowed any visitors. She could not hold the dying person's hand, as she was required to attend to the patient with critical needs next door. In addition, she was "forced" to deny the dying person's relatives access to the unit.

How would you assess this situation? Please explain your answer. Is there anything you would like to share in this context?

Narrative prompt:
What do you think could be a way to identify symptoms of the "Moral Injury" phenomenon in practice?

Possible follow-up questions:

- Do you believe that discussing situations involving Moral Injury in supervision sessions could help identify it?
- In your opinion, should something be actively addressed in this regard? If so, what?

Interview Conclusion

Is there anything else you would like to talk about—anything that is important to you that we haven't discussed yet?

Instructions: Thank you very much for the conversation and for answering so openly.

Stop the recording

How are you feeling now? How was the interview experience for you?
